# The genus *Rhynchobanchus* Kriechbaumer in China, with descriptions of a new species and first record of the genus from Oriental region (Hymenoptera, Ichneumonidae, Banchinae)

**DOI:** 10.3897/zookeys.752.23884

**Published:** 2018-04-23

**Authors:** Ze-Jian Li, Tao Li, Jun Yan, Mao-Ling Sheng

**Affiliations:** 1 Key Laboratory of Zoological Systematics and Evolution, Institute of Zoology, Chinese Academy of Sciences, Beijing 100101, China; 2 General Station of Forest Pest Management, State Forestry Administration, Shenyang, Liaoning, 110034, China; 3 Postdoctoral Work Station, Lishui Academy of Forestry, Lishui, 323000, China

**Keywords:** Banchini, China, key, new species, Oriental Region, Palaearctic Region, *Rhynchobanchus*

## Abstract

Six species and two subspecies of the genus *Rhynchobanchus* Kriechbaumer, 1894 are reported from China, of which one, *Rh. flavomaculatus* Sheng, **sp. n.**, is a new species and the first record of the genus from the Oriental Region. *Rhynchobanchus flavopictus orientalis* Kuslitzky, 2007 is a new Chinese record. A key to the species of *Rhynchobanchus* occurring in China is provided.

## Introduction

*Rhynchobanchus* Kriechbaumer, 1894, belonging to the tribe Banchini of the subfamily Banchinae (Hymenoptera: Ichneumonidae) and comprising seven species (Yu et al. 2016), is only known from the Palaearctic Region (Kriechbaumer 1894; Meyer 1927; Sheng et al. 1995, 1997; Kuslitzky 2007; Kim et al. 2012; Sheng and Sun 2014, Watanabe 2016). Four species restricted to the Eastern Palaearctic Region, one to the Western Palaearctic Region, and two species are trans-Palaearctic. The diagnostic characters of the genus were most recently revised by Sheng et al. (2014).

The aim of this study is to revise all available materials of *Rhynchobanchus* from China, describe one new species from the Oriental part of China, and provide an identification key to species occurring in China.

## Materials and methods

Type specimens were collected using entomological sweep nets in the forest of Tianmu Mt., Lin’an, Zhejiang Province and Shaoyang, Hunan Province (China). Other Chinese specimens from the collections in the Insect Museum, General Station of Forest Pest Management, State Forestry Administration, People’s Republic of China (GSFPM) were checked.

The holotype locality is a forest comprised of mixed deciduous angiosperms and evergreen conifers, mainly including *Liquidambar formosana* Hance, *Aphananthe aspera* (Thunb.), *Acer* spp., *Quercus* sp., *Castanea* spp., *Elaeagnus pungens* Thunb., *Rosa multiflora* Thunb., *Euscaphis japonica* (Thunb.) Dipppel, *Lindera glauca* (Sieb. et Zucc.) Bl., *Pinus massoniana* Lambert, *Cryptomeria japonica* (Linn.f.) D. Don, and *Metasequoia glyptostroboides* Hu et Cheng.

The photos of *Rh. flavopictus orientalis* Kuslitzky, 2007 (holotype), *Rh. bicolor* Kriechbaumer, 1894 identified by Townes, and *Rh. flavopictus* Heinrich, 1937 identified by Kuslitzky, deposited in the Zoological Institute of the Russian Academy of Sciences, St. Petersburg, Russia (ZISP), were compared to the new species.

Images were taken using a stereomicroscope Leica M205A with a LAS Montage MultiFocus. Morphological terminology is mostly based on Gauld (1991).

All examined material, including type specimens of the new species, is deposited in **GSFPM**.

## Taxonomy

### Rhynchobanchus

Taxon classificationAnimaliaHymenopteraIchneumonidae

Kriechbaumer, 1894

#### Type-species.

*Rhynchobanchus bicolor* Kriechbaumer.

#### Diagnosis.

*Rhynchobanchus* can be distinguished from all other genera of Banchini by a combination of the following characters: Antennae long and slender; apical margin of clypeus with a median notch (Fig. 2); upper tooth of mandible very wide, its apex oblique and concave, subdivided into two teeth (Fig. 2); lower tooth of mandible pointed; epicnemial carina absent (Fig. 5); propodeum short, without carinae (Fig. 6); fore wing with areolet receiving 2m-cu near center (Figs 7, 15); nervulus distal of 1/M by at least 0.5 its length; tarsal claws strongly pectinate; ovipositor sheath very short, slightly projecting beyond apex of metasoma (Fig. 9).

#### Host.

Unknown.

#### Key to females of species of *Rhynchobanchus* known in China

**Table:** 

1	Hindwing vein 1-cu almost disappeared (basal end of 2-Cu almost touching M+Cu). Areolet pentagonal (Fig. 15). Facial orbits with yellow longitudinal streak. Tergites black; posterior three sternites red to reddish brown. Scutellum yellow	***Rh. rufus* Sheng & Sun**
–	Hindwing vein 1-cu distinct, approximately 0.2 as long as cu-a. Areolet quadrangular, if pentagonal, at least median tergites red. Others not entirely as above	**2**
2	Propodeum very rough. Ovipositor sheath evidently reaching past tip of metasoma. Antenna black, or slightly brown ventrally. Mesosoma black, or at most anterolateral portion of mesoscutum with small yellow spots. Tergites black, or at most posterolateral portion of tergites 1 to 4 with small yellow spots	***Rh. niger* Sheng**
–	Propodeum smooth or slightly rough, with distinct punctures or winkles. Ovipositor sheath at most reaching tip of metasoma. Antennae light in color, at least ventral profile red or reddish brown. Mesosoma with yellow spots. Scutellum yellow. Median portion of metasoma reddish brown, or at least apical lateral portion light in color	**3**
3	Longest spur of mid tibia approximately 0.8 times as long as first tarsomere. Body predominantly yellow with brown markings (Fig. 1); median portion of frons, inverted triangular median stripe of mesoscutum, transverse groove in front of propodeum, median spots of first tergite black	***Rh. flavomaculatus* sp.n.**
–	Longest spur of mid tibia at most 0.7 times as long as first tarsomere. Body predominantly black with many yellow or brown spots	**4**
4	Flagellum reddish brown, its ventral profile slightly dark-reddish brown. Face reddish brown, or with small black spot. Basal portions of tergites 1 to 5 black, apical portions reddish brown; remainder of tergites almost entirely reddish brown. Hind tarsus dark reddish brown	***Rh. flavopictus flavopictus* Heinrich**
–	Flagellum black, or dorsal profile black, ventral red. Face black. Tergites black, or median tergites red	**5**
5	Tergite 2 as long as apical width. Propodeum and hind tarsus entirely black. Tergites black, at most lateral portions of median tergites with small yellow spots	***Rh. flavopictus orientalis* Kuslitzky**
–	Tergite 2 longer or shorter than apical width. Apical portion of propodeum yellowish brown. Hind tarsus brown or darkish brown. Tergites 2 to 4 reddish brown	**6**
6	Areolet pentagonal. Spiracle of first tergite strongly convex. First tergite 2.4 times as long as apical width, spiracle strongly convex (Fig. 12). Mediolateral portions of median tergites with yellow spots	***Rh. maculicornis* Sheng, Liu & Wang**
–	Areolet quadrangular. First tergite 2.2 times as long as apical width, spiracle almost not convex (Fig. 13). Median tergites without yellow spot	***Rh. minomensis* (Uchida)**

### Rhynchobanchus flavomaculatus

Taxon classificationAnimaliaHymenopteraIchneumonidae

Sheng sp.n.

http://zoobank.org/87F55DB7-362C-4FE9-B7C6-25DA7EFA2E55

Figures 1–5 , 6–9

#### Material.

Holotype, Female, **CHINA**: Kaishanlaodian, 1106 m, 30°32'N, 119°43'E, West Tianmu Mt., Lin’an, Zhejiang Province, 28 April 2017, leg. Ze-Jian Li, Meng-Meng Liu & Kai-Wen Gao. Paratypes: 1 female, Yun Mt., 1380m, 26°38'N, 110°37'E, Wugang, Shaoyang, Hunan Province, 18 April 2011, leg. Ze-Jian Li. 1 female, same data as holotype, but 8 April 2013, leg. Li-Wei Qi & Biao Chu. 1 male, same data as holotype, but 14 April 2014, leg. Hai-Yan Nie & Ping Hu. 2 females, same data as holotype, but 23/24 April 2014, leg. Ting-Ting Ji.

#### Diagnosis.

**Figures 1–5.**
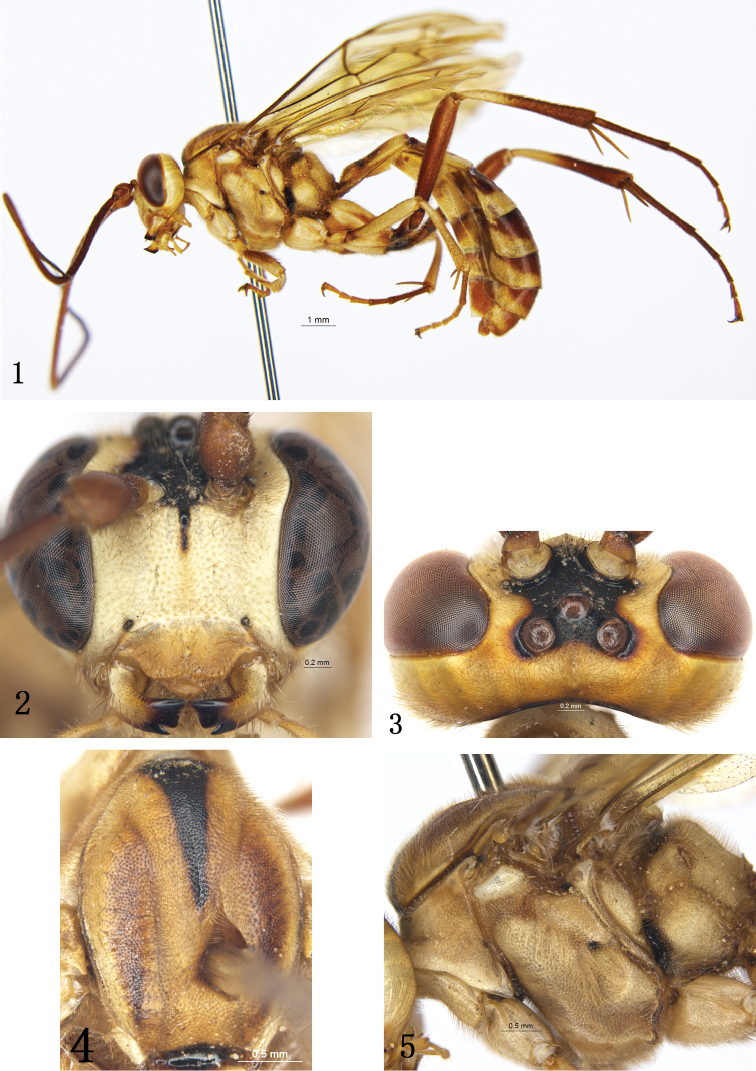
*Rhynchobanchus flavomaculatus* sp. n. Holotype. Female. **1** Habitus, lateral view **2** Head, anterior view **3** Head, dorsal view **4** Mesoscutum **5** Mesosoma, lateral view.

Body yellowish brown with large irregular yellowish white spots (Fig. 1). Face (Fig. 2) finely granulose, with sparse fine punctures and yellowish brown setae, near upper margin with a small median tubercle. Inner orbits of compound eyes distinctly emarginate opposite antennal sockets, with regular yellowish brown setae. Posteromedian portion of vertex (behind ocellar triangle) with weak longitudinal groove (Fig. 3). Lower-posterior corner of mesopleuron with strong swelling (Fig. 5). Wings (Fig. 7) yellowish brown, semi-hyaline. Longer spur of mid tibia approximately 0.8 times as long as first tarsomere. Tergite 1 about 2.3 times as long as apical width, distinctly convex basal of spiracle.

#### Description.

Female (Fig. 1). Body length 15.6 to 17.1 mm. Forewing length 11.3 to 12.9 mm.

*Head*. Face (Fig. 2) approximately 1.7 times as wide as long, finely granulose, with sparse fine punctures and yellowish brown setae, median portion longitudinally convex; near upper margin with a small median tubercle. Clypeus approximately 2.5 times as wide as long, finely granulose, with indistinct, sparse brown setae; apical margin with distinct median emarginate. Mandible particularly strong, basal portion with sparse brown setae. Malar area finely granulose, approximately 0.5 times as long as basal width of mandible. Inner orbits of compound eyes distinctly emarginate opposite antennal sockets. Gena broad, evenly convergent backwardly, with dense, fine punctures and yellowish brown setae. Vertex (Fig. 3) with yellowish brown setae and weak median longitudinal groove. Ocellar triangle with distinct punctures. Postocellar line approximately 1.3 times as long as oculo-ocellar line. Lower median portion of frons slightly concave, with fine arched wrinkles; lateral portion with dense yellowish brown setae. Lateral margin of antennal socket with small tubercle. Antenna with 55 to 58 flagellomeres, ratio of length from first to fifth flagellomeres: 2.1:1.1:1.0:1.0:1.0. Occipital carina complete.

**Figures 6–9.**
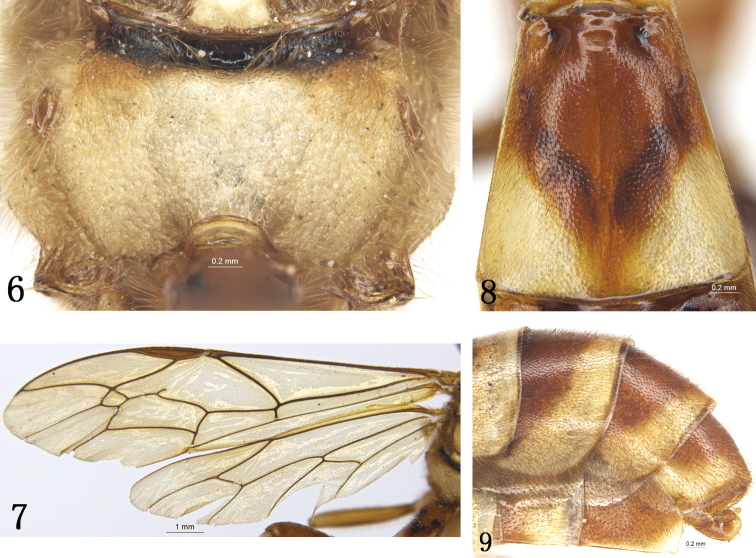
*Rhynchobanchus flavomaculatus* sp. n. Holotype. Female. **6** Propodeum **7** Wings **8** Tergite 2, dorsal view **9** Apical portion of metasoma, lateral view.

*Mesosoma*. Pronotum (Fig. 5) with granulose texture and dense yellowish brown setae; Epomia absent. Mesoscutum (Fig. 4) evenly convex, anterior and lateral portions with distinct dense punctures and brown setae, posteromedian portion with fine, indistinct punctures. Notaulus weak. Scutoscutellar groove smooth, shiny. Scutellum almost rounded convex, with fine dense punctures and long brown setae. Postscutellum evenly convex, with fine punctures. Mesopleuron (Fig. 5) granulose with even fine punctures and brown setae; speculum very small, with fine punctures; mesopleural fovea small, shallow, smooth. Lower-posterior corner of mesopleuron strongly convex. Metapleuron evenly convex, with texture as that of mesopleuron; lower posterior portion with indistinct oblique wrinkles. Submetalpeural carina complete, strong, anterior portion strongly raised. Sulcus between postscutellum and propodeum deep, shiny, with short longitudinal wrinkles. Propodeum (Fig. 6) short, slightly oblique; weakly, finely reticulate, with dense yellowish brown setae; spiracle convex, oblique elongate, 3.0 times as long as width, located at basal 0.3.

*Wings* (Fig. 7). Yellowish brown, semi-hyaline. Forewing with vein 1cu-a distal of 1/M, distance between them approximately 0.5 times as long as 1cu-a. Ramulus long. Areolet pentangle, receiving 2m-cu approximately at basal 0.4, vein 3rs-m slightly longer than vein 2rs-m. 2-Cu 1.5 times as long as 2cu-a. Hindwing vein cu-a strongly reclivous, 1-cu 0.15 times as long as cu-a.

*Legs*. Dorsal profile and apical portion of tibia with uneven thorns. Longer spur of mid tibia 0.8 times as long as first tarsomere. Ratio of length of hind first to fifth tarsomeres 5.8:2.5:2.0:1.0:1.5.

*Metasoma*. Tergite 1 about 2.3 times as long as apical width, distinctly convex basal of spiracle, with fine punctures and yellowish brown short setae, apical margin smooth; glymma deep, small; spiracle small, convex, almost circular, located at basal 0.3. Tergite 2 (Fig. 8) approximately as long as apical width, indistinctly reticular, with dense punctures and yellowish brown short setae; basal median portion transversely convex, smooth; thyridium distinct, almost smooth. Tergite 3 weakly shining, with dense fine punctures and yellowish brown setae, apical portion slightly compressed. Fourth and subsequent tergites compressed, with fine punctures and yellowish brown setae. Ovipositor sheath broad, not reaching to apex of metasoma.

*Colour pattern* (Fig. 1). Yellowish brown with large irregular yellowish white spots, except following: mandibular teeth, median portion of frons, ocellar triangle, line along occipital carina, anteromedian narrow reversed triangular spot, scutoscutellar groove, anterior portion of mesosternum, anterior margin of metapleuron, anterior transverse groove of propodeum, front and inner profiles of hind coxa, trochanter (dorsal and apical portions reddish brown), median portion of tergite 1 irregularly dark brown to black. Antenna except apical portion brown to dark brown, main portion of front femur, ventral profile of mid femur slightly; apical margin of hind coxa, hind trochantellus, hind femur, hind tibia except basal half yellowish brown, hind tarsus; tergites 2 and 3 except apical triangular spots yellow to brown, tergites 3 to 7 except lateral and apical margin yellow to yellowish brown; tergite 8, ovipositor sheath reddish brown to dark reddish brown. Pterostigma brown. Veins brown to dark brown.

#### Male.

Body length approximately 13.1 mm. Forewing length approximately 10.0 mm. Antenna with 55 flagellomeres. Lateral longitudinal stripes of mesoscutum, transverse stripe beneath subalar ridge black brown to brown. Pterostigma yellowish brown. Otherwise similar to female.

#### Distribution.

CHINA: Hunan, Zhejiang.

#### Remarks.

This new species is similar to *Rh. maculicornis* Sheng et al., 1995, but can be distinguished from the latter by the following combination of characters: lower-posterior corner of mesopleuron with strong convexity; gena, vertex, mesopleuron and mesosternum yellow; and propodeum yellowish white. *Rhynchobanchus maculicornis* has lower-posterior corner of mesopleuron slightly convex; gena, vertex, mesopleuron and mesosternum black; basal portion of propodeum black, median reddish brown and apical yellow.

#### Etymology.

The name of the new species is derived from Latin words “flavi” (yellow) and “maculatus” (macula) after its body with large irregular yellowish spots.

### Additional records for Chinese species of *Rhynchobanchus*

#### Rhynchobanchus flavopictus flavopictus

Taxon classificationAnimaliaHymenopteraIchneumonidae

Heinrich, 1937

Figure 10

##### Specimen examined.

1 female, CHINA: Xinbin, Liaoning Province, 28 May 1994, leg. Mao-Ling Sheng (GSFPM).

##### Distribution.

China, Germany, Italy, Italy, Poland, Russia, United Kingdom.

**Figure 10.**
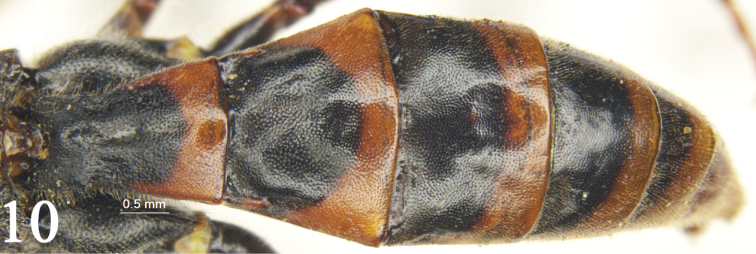
*Rhynchobanchus flavopictus flavopictus* Heinrich, 1937. Female. Metasoma, dorsal view.

#### Rhynchobanchus flavopictus orientalis

Taxon classificationAnimaliaHymenopteraIchneumonidae

Kuslitzky, 2007

Figure 11

##### Specimen examined.

1 female, CHINA: Benxi, Liaoning Province, 6 June 2007, leg. Mao-Ling Sheng (GSFPM). **New record for China**.

##### Distribution.

China, Russia.

**Figure 11.**
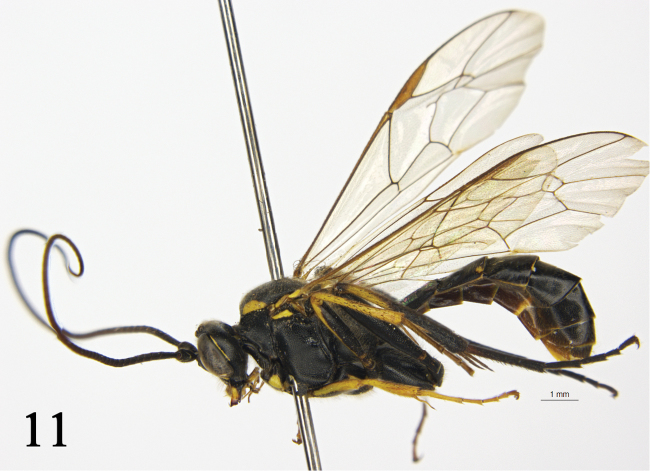
*Rhynchobanchus flavopictus orientalis* Kuslitzky, 2007. Female. Habitus, lateral view.

#### Rhynchobanchus maculicornis

Taxon classificationAnimaliaHymenopteraIchneumonidae

Sheng, Liu & Wang, 1995

Figure 12

##### Specimens examined.

1 male (holotype), CHINA: Benxi, Liaoning Province, 12 May 1985, leg. Shou-Lin Liu (GSFPM). 3 males (paratypes), same data as holotype (GSFPM). 1 male: Laotudingzi, Huanren, Liaoning Province, 25 May to 9 June 2011, IT (GSFPM). 1 male: Chagou, Haicheng, Liaoning Province, 15 May 2015, leg. Tao Li (GSFPM). 2 females, 2 males: Benxi, Liaoning Province, 30 May 2016, leg. Shu-Ping Sun (GSFPM). 5 males: Benxi, Liaoning Province, 16 May 2017, leg. Mao-Ling Sheng (GSFPM). 1 male: Kuandian, Liaoning Province, 18 May 2017, leg. Tao Li (GSFPM).

##### Distribution.

China (Liaoning Province).

**Figure 12.**
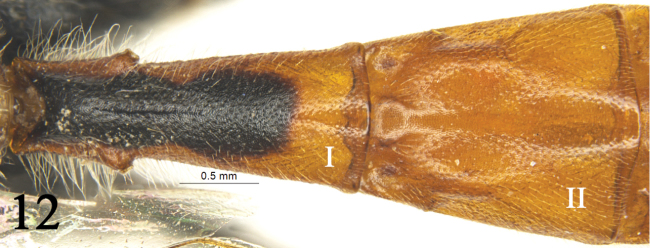
*Rhynchobanchus maculicornis* Sheng, Liu & Wang, 1995. Female. Tergites I–II, dorsal view.

#### Rhynchobanchus minomensis

Taxon classificationAnimaliaHymenopteraIchneumonidae

(Uchida, 1933)

Figure 13

##### Specimens examined.

1 female, CHINA: Xinbin, Liaoning Province, 29 May 1994, leg. Mao-Ling Sheng (GSFPM). 2 females: Baishilazi, Kuandian, Liaoning Province, 26 May to 9 June 2011, IT (GSFPM).

##### Distribution.

China, Japan, Korea, Russia.

**Figure 13.**
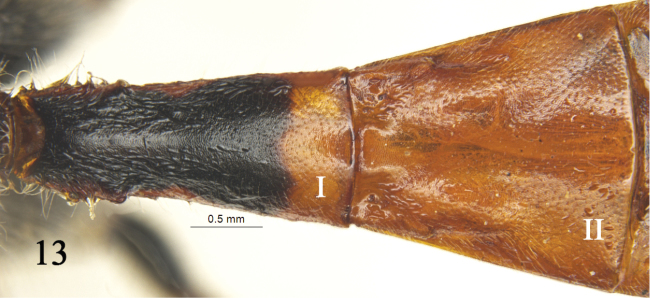
*Rhynchobanchus minomensis* (Uchida, 1933). Female. Tergites I–II, dorsal view.

#### Rhynchobanchus niger

Taxon classificationAnimaliaHymenopteraIchneumonidae

Sheng, Li & Pang, 1997

Figure 14

##### Specimens examined.

1 female (holotype), CHINA: Xinbin, Liaoning Province, 28 May 1994, leg. Mao-Ling Sheng (GSFPM). 28 males (paratypes), id., but 28/ 29 May, 1994. 2 males (paratypes), CHINA, Shenyang, Liaoning Province, 8 May, 1994. 1 female: Kuandian, Liaoning Province, 4 June 2001, leg. Shu-Ping Sun (GSFPM). 1 female: Shenyang, Liaoning Province, 6 May 2002, leg. Mao-Ling Sheng (GSFPM). 1 female: Benxi, Liaoning Province, 27 May 2006, leg. Chun-Tian Zhang (GSFPM). 1 female: Kuandian, Liaoning Province, 7 June 2007, leg. Shu-Ping Sun (GSFPM). 1 female: Laotudingzi, Huanren, Liaoning Province, 25 May 2011, Mao-Ling Sheng (GSFPM).

##### Distribution.

China (Liaoning Province).

**Figure 14.**
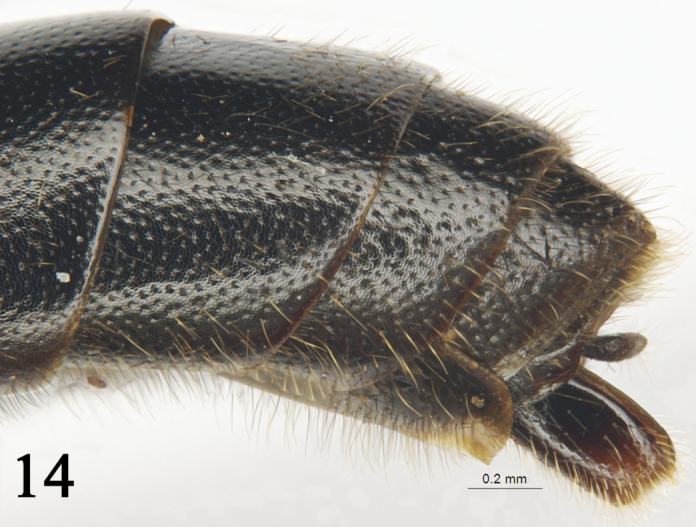
*Rhynchobanchus niger* Sheng, Li & Pang, 1997. Holotype. Female. Apical portion of metasoma, lateral view.

#### Rhynchobanchus rufus

Taxon classificationAnimaliaHymenopteraIchneumonidae

Sheng & Sun, 2014

Figure 15

##### Specimens examined.

1 female, CHINA: Shenyang, Liaoning Province, 3 May 2003, leg. Mao-Ling Sheng (GSFPM). 1 female (holotype): Kuandian, Liaoning Province, 6 June 2007, leg. Shu-Ping Sun (GSFPM). 1 female (paratype): Xinbin, Liaoning Province, 28 May, 1994, leg. Mao-Ling Sheng (GSFPM). 1 female (paratype): Huanren, Liaoning Province, June, 1996 (GSFPM). 3 females (paratypes): Kuandian, Liaoning Province, 6 to 8 June 2007, leg. Shu-Ping Sun & Mao-Ling Sheng (GSFPM). 1 female (paratype): Tieshashan, 900m, Benxi, Liaoning Province, 12 June 2011, leg. Ya-Nan Tang & Ying Zhang (GSFPM). 1 female (paratype), id., 19 June 2011, Tie-Fei Zhao & Ying Yang. 1 female (paratype): Benxi, Liaoning Province, 19 June 2013, IT (GSFPM). 1 female: Benxi, Liaoning Province, 12 June 2015, leg. Mao-Ling Sheng (GSFPM).

##### Distribution.

China (Liaoning Province).

**Figure 15.**
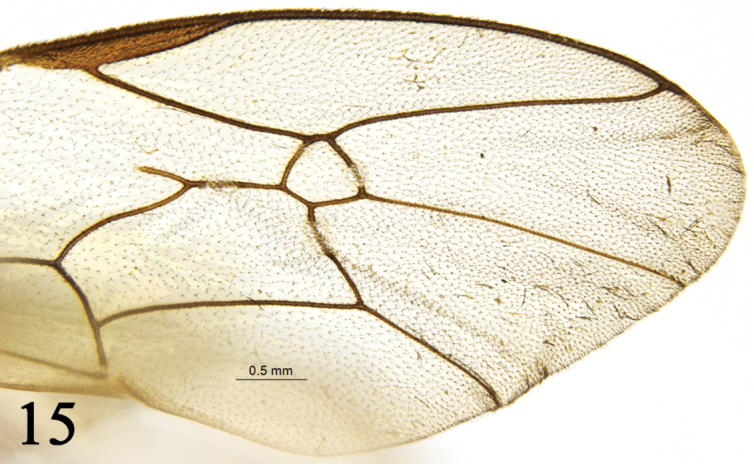
*Rhynchobanchus rufus* Sheng & Sun, 2014. Holotype. Female. Apical portion of forewing.

## Conclusions

There are now eight known species of *Rhynchobanchus* Kriechbaumer in the world, of which one is only known from the Oriental Region, and seven species are known from the Palaearctic Region. Six species have been known from China. Hitherto, there are no host records.

## Supplementary Material

XML Treatment for Rhynchobanchus

XML Treatment for Rhynchobanchus flavomaculatus

XML Treatment for Rhynchobanchus flavopictus flavopictus

XML Treatment for Rhynchobanchus flavopictus orientalis

XML Treatment for Rhynchobanchus maculicornis

XML Treatment for Rhynchobanchus minomensis

XML Treatment for Rhynchobanchus niger

XML Treatment for Rhynchobanchus rufus
